# In Katrina’s Wake

**DOI:** 10.1289/ehp.114-a32

**Published:** 2006-01

**Authors:** John Manuel

Hurricane Katrina has been called the most devastating natural environmental calamity in U.S. history. Visitors to the scene say the destruction is worse than anyone can imagine. Scientists also say that some perceived health threats have been overblown and others understated. Months after Katrina roared into the Gulf Coast, the environmental health implications of the storm are still being assessed.

Katrina presented residents of the Gulf Coast with a bewildering array of environmental health hazards. Aside from standing floodwater, hazards included a lack of potable water, sewage treatment, and electricity; chemical spills; swarms of insects (with anecodotal accounts of vermin and hungry domestic dogs); food contamination; disrupted transportation; mountains of debris; buildings damaged and destroyed; rampant mold growth; tainted fish and shellfish populations; and many potential sources of hazardous waste. Some impacts, such as deaths from drowning and injuries from cleaning up debris, have been relatively easy to determine. Others, such as post-traumatic stress disorder from the loss of homes and loved ones, may never be fully quantified.

In the weeks following the storm, federal agencies such as the NIEHS, the Centers for Disease Control and Prevention (CDC), and the Environmental Protection Agency (EPA), as well as state environmental and public health agencies, sent scientists to the region to begin assessing the environmental and human health impact of the disaster. Much of what they found was presented on October 20 at a meeting of the National Academies Institute of Medicine’s Roundtable on Environmental Health Sciences, Research, and Medicine (commonly known as the EHSRT), supported by the NIEHS, the CDC, the EPA, Exxon-Mobile Corporation, the American Chemistry Council, and the Brita Water Research Institute. Still more information continues to emerge today. And much simply remains to be seen.

## Katrina Hits

Katrina, rated as a Category 4 hurricane on the Saffir-Simpson scale, made landfall near New Orleans on 29 August 2005. Wind damage extended as far as 150 miles inland. Heavy rain battered the area, and the storm surge—measuring as high as 30 feet and sweeping several miles inland—breached several levees intended to protect New Orleans from the waters of Lake Pontchartrain. Water poured through the breaks in the days following the storm, covering approximately 80% of the city with water as deep as three meters. The American Red Cross estimates that more than 354,000 homes along the Gulf Coast were destroyed or damaged beyond repair by Katrina and, a month later, Hurricane Rita. Hundreds of small manufacturers or businesses using chemicals or fuels also were impacted.

Flooding, wind, and waves caused major damage to buildings and infrastructure whose integrity is key to the environmental health of the local citizenry. The EPA estimated that more than 200 sewage treatment plants in Louisiana, Mississippi, and Alabama were affected, with almost all the plants around New Orleans knocked out of action. Loss of power meant lift stations (which pump sewage uphill) could not work, causing sewage to overflow into houses and streets.

The region struck by Katrina and Rita is home to a large number of oil refineries and chemical plants. Prior to Katrina, the EPA had identified nearly 400 sites in the affected area as possibly needing cleanup because of their potential impact on human health. Following the storm, the U.S. Coast Guard reported numerous oil spills from refineries and tank farms in South Louisiana. A story in the September 30 *Boston Globe* reported that Katrina damaged 140 oil and gas platforms in the Gulf of Mexico, 43 seriously, including some that floated away or sank.

Across the Gulf Coast, more than 1.5 million people evacuated as the storm approached. More than 100,000 stayed behind in New Orleans, unwilling or unable to leave. As New Orleans flooded, thousands waded through chest-deep floodwaters to reach shelters or higher ground. Thousands more remained trapped in homes, hospitals, and nursing homes. Conditions in shelters rapidly became unsanitary. Many people were exposed to the elements for five days or more, living with little or no food, drinking water, or medicine. As of December 5, the death toll was reported at 1,071 in Louisiana, 228 in Mississippi, 14 in Florida, 2 in Alabama, and 2 in Georgia.

## First Response

Numerous federal, state, and local agencies, as well as private individuals and relief groups, swung into action in the wake of the storm. Troops from the U.S. Army, Coast Guard, and National Guard as well as state and local officials and private citizens rescued those they could. The Federal Emergency Management Agency (FEMA) was assigned the lead in disaster relief planning and administration, including provision of emergency food and shelter and contracting for debris removal. The Department of Health and Human Services (DHHS) declared a public health emergency in the Gulf states and directed the CDC to take appropriate action. The CDC deployed more than 600 professionals into the disaster zone, including specialists in public health nursing, occupational safety and health, laboratory science, medicine, epidemiology, sanitation, environmental health, disease surveillance, public information, and health risk communication.

The CDC also joined with the EPA to set up a joint task force to conduct an environmental health needs and habitability assessment to identify critical public health issues for the reinhabitation of New Orleans. This city was unique among the areas hit in that it was the only one left with standing water. Major urban areas in Mississippi and Alabama, while devastated, did not remain flooded.

In advance of the storm’s arrival, the EPA had predeployed teams to the area, with the mission of guiding debris disposal, assisting in the restoration of drinking and wastewater treatment systems, and containing hazardous waste spills. Immediately after the storm, these teams used their 60 watercraft to help search-and-rescue efforts, rescuing about 800 people, according to EPA administrator Stephen Johnson. Five days after the storm, the EPA began testing floodwaters in New Orleans for biological and chemical contamination.

In coordination with the Louisiana Department of Environmental Quality (LDEQ), the EPA analyzed floodwaters for more than 100 hazardous pollutants such as volatile and semivolatile organic compounds, metals, pesticides, herbicides, and polychlorinated biphenyls. They also tested for biological agents such as *Escherichia coli*. Their testing revealed “greatly elevated” levels of *E. coli*, as much as ten times higher than EPA’s recommended levels for contact. According to the EPA, agency scientists found levels of lead and arsenic at some sites in excess of drinking water standards—a potential threat given the possibility of hand-to-mouth exposure. The EPA posted these and other findings on its Hurricane Response 2005 website (http://www.epa.gov/katrina/), created after the storm.

Shortly after the hurricane struck, the U.S. Coast Guard began working with the EPA, the Louisiana state government, and private industries to identify and recover spilled oil along the coast. The team identified 6 major, 4 medium, and 134 minor spills totaling 8 million gallons. One of the most notorious spills occurred at the Murphy Oil Company plant, which dumped more than 25,000 barrels of oil into the streets of Chalmette and Meraux, Louisiana. As of December 7, the Coast Guard reported the recovery of 3.8 million gallons, with another 1.7 million evaporated, 2.4 million dispersed, and 100,000 onshore.

Meanwhile, the NIEHS was joining with Duke University Medical Center, the NIH, and the CDC to provide assistance with relief and recovery operations along the Gulf Coast, as well as working at home to establish a web-site on environmental health issues related to Katrina [for more information, see “NIEHS Responds to Katrina,” p. A28 this issue].

## Floodwater Hazards

Kevin Stephens is director of the New Orleans Department of Health. He was in charge of interpreting the EPA data and advising citizens and responders about the health hazards presented by the floodwaters. “I struggled every day to determine what [the data] meant and what to tell our health workers and the public,” he says. “What does ‘not an immediate health hazard’ mean when you have people wading through the water? What does ‘not in excess of drinking water standards’ mean? Is it a danger if you get your hands wet and touch your mouth?” Journalists claimed the floodwaters were a “toxic gumbo” of dangerous chemicals and microbes, raising fears that any contact was a health threaten.

These concerns prompted a team of scientists led by John Pardue, director of the Louisiana Water Resources Research Institute at Louisiana State University (LSU), to conduct its own study of the New Orleans floodwaters. The report, published 15 November 2005 in *Environmental Science & Technology*, stated categorically that, contrary to claims in the media, the floodwater was not a “toxic soup.”

“Chemical oxygen demand and fecal coliform bacteria were elevated in surface flood-water, but typical of stormwater runoff in the region,” the report said. “Lead, arsenic, and in some cases chromium exceeded drinking water standards, but with the exception of some elevated lead concentrations were generally typical of stormwater.” The LSU study also found only low concentrations (less than 1%) of benzene, toluene, and ethylbenzene even in places where there was a visible oil sheen. “Collectively, these data indicate that Katrina floodwater is similar to normal stormwater runoff, but with somewhat elevated lead and VOC concentrations,” the report concluded.

However, the LSU study was limited to two areas within the city of New Orleans, and the authors warned that conditions could be different elsewhere, particularly in Lake Pontchartrain, where floodwaters were being pumped. LSU and the University of Colorado are currently conducting studies of Lake Pontchartrain looking for a wide range of pathogens. The Colorado team is measuring aerosols created by pumping floodwater into the lake, while the LSU team is analyzing the lake water itself.

## More Water Hazards

Still other threats were posed by water. As of December 9, the EPA reported that 99% of the waste treatment and water supply systems were back online, but some had been out of operation for weeks. At the October 20 EHSRT, Howard Frumkin, director of the National Center for Environmental Health and Agency for Toxic Substances and Disease Registry (NCEH/ATSDR), said that despite the percentage of sewage treatment plants already online at that point, the danger wasn’t over. “We have no guarantees that sewage being flushed is getting to treatment plants,” he said. “Raw sewage is going into the Mississippi River.”

Though most water supply systems may be functioning again, the safety of distribution lines that were flooded can’t yet be ensured either. “There are possible changes in pipe ecology due to the intrusion of contaminants,” said Frumkin. “And we have additional concerns for homes on wells.” Louisiana officials speaking at the roundtable said there are dozens of community water systems and tens of thousands of private wells that need to be tested for contamination.

Standing water poses a different threat, serving as a breeding ground for bacteria and mosquitoes. Even prior to Katrina, Louisiana had the highest number of reported cases of West Nile virus (66) of any state in the union, according to the CDC. West Nile virus can be transmitted to humans via mosquito bites, and the warm, wet weather following the storm was ideal for breeding of mosquitoes. The U.S. Air Force sprayed areas of standing water with pesticides to kill mosquito larvae. The CDC reported on its Update on CDC’s Response to Hurricanes website that postspraying surveillance at ten sites found a 91% reduction in total mosquito density compared to prespraying surveillance results [for more information on this website, see the EHPnet article, p. A27 this issue].

The Gulf Coast is also known for the presence of the bacterium *Vibrio vulnificus*. This relative of the pathogen that causes cholera thrives in brackish waters in warmer times of the year. Humans may become infected by eating contaminated seafood or through open wounds exposed to water. While not harmful to individuals in good health, it can be fatal to those with liver damage. Health officials at the roundtable reported counting 22 cases of illness induced by *V. vulnificus* following the storm, including 5 deaths.

In late September, the EPA launched the Ocean Survey Vessel *Bold* to conduct water quality testing in the river channels and nearshore waters of the Mississippi Delta. The agency monitored 20 areas to determine whether fecal pollution from flooded communities had spread into these waters. All 20 monitoring stations showed that, at the time, the water was safe for primary contact, including swimming. The EPA said on its website, however, that the data “should not be used to assess the safety of consuming raw or undercooked molluscan shellfish.”

In the wake of the storm, Louisiana, Mississippi, and Alabama closed their shellfishing waters until testing could be done. On December 8, the three states issued a joint press release saying that fish and shellfish samples collected and analyzed since the hurricanes “show no reason for concern about the consumption of Gulf seafood.” Louisiana and Alabama subsequently reopened their waters, while Mississippi’s oyster reefs remain closed pending additional studies.

## Toxicants in Sediment and Air

Health officials also anticipated a threat from contaminated sediment in the days and weeks following the storm. As floodwaters were pumped out of inundated areas, a dark sludge was found coating buildings, land, and pavement. *E. coli* was detected at elevated levels in many sediment samples taken from around New Orleans, implying the presence of fecal bacteria. The EPA has no standards for determining human health risks from *E. coli* in sediment, but warned people to limit exposure, and if exposed, to wash skin with soap and water.

The EPA was concerned, too, about the region’s Superfund sites, which include former dump sites of pesticides and dioxins. The EPA identified 54 Superfund sites in the affected area. Officials worried that at least some of these sites might have been compromised, releasing toxic chemicals into the land or water. Johnson reported at the EHSRT that as of October 20, the EPA had visually inspected all of the sites and sampled many. As of December 5, the EPA’s posted test results for these sites indicated that none were compromised in a way that would present a human health hazard.

Elsewhere, as late as November 20, chemical testing of sediment samples in Louisiana’s Orleans and St. Bernard Parishes indicated the continued presence of petroleum. However, the EPA’s website states that exposures of emergency responders at these levels are not expected to cause adverse health effects as long as the proper personal protective equipment is worn, such as gloves and safety glasses. Volatile and semivolatile organic compounds, pesticides, and metals including aluminum were found, but at levels below what the ATSDR and CDC consider to be immediately hazardous to human health. However, the site continues, “EPA and ATSDR/CDC continue to recommend that residents avoid all contact with sediment deposited by floodwater, where possible, due to potential concerns associated with long-term skin contact.”

The Natural Resources Defense Council (NRDC) and a host of local environmental groups paint a darker picture of the contamination situation. In a December 1 press release, the NRDC stated that tests it had conducted revealed “dangerously high levels” of industrial chemicals and heavy metals in the sediment covering much of New Orleans. For example, tests found arsenic levels in some neighborhoods that exceeded EPA safety limits by a factor of 30.

“We found arsenic and other cancer-causing contaminants in sediment all across the entire city,” said Monique Hardin, co-director of the New Orleans–based Advocates for Human Rights, at an NRDC press briefing. “We also found hot spots where there were some nasty surprises, such as banned pesticides.” The groups urged the EPA to begin cleaning up or removing contaminated topsoil across the city and to conduct further testing in certain neighborhoods.

The NRDC also challenged the EPA’s assertion that the flooded Superfund sites posed no threat. The December 1 press release stated that NRDC’s own assessment of one of these sites, the New Orleans Agricultural Street Landfill Superfund Site, showed “visible leachate emerging from the site and spreading across the street and onto a local senior center’s property. Sediment testing at this site found contamination as much as 20 times higher than the EPA soil cleanup standards for four [polycyclic aromatic hydrocarbons].”

LDEQ toxicologist Tom Harris responded in press reports that the NRDC’s findings were fundamentally flawed because arsenic levels are naturally above the EPA’s residential standard in Louisiana and elsewhere. “I have never personally seen soil samples come back below the residential screening level for arsenic,” Harris told PlanetArk World Environmental News on December 5. “It’s a naturally occurring [element] you can find everywhere.” The state of Louisiana and the EPA continue to perform testing of sediment to determine when to give an all-clear to residents with respect to exposure to sediment.

The EPA has also addressed concerns about air quality in the Gulf region. According to Johnson, most of the agency’s stationary air quality monitors were knocked out by Katrina. The EPA reinstalled the stationary monitors and employed their Airborne Spectral Photometrics Environmental Collection Technology to undertake airborne monitoring. The EPA also employed two Trace Atmospheric Gas Analyzer buses, self-contained mobile laboratories capable of continuous real-time sampling and analysis.

Air samples were tested for volatile priority pollutants such as benzene, toluene, and xylene, which are commonly found in gasoline, as well as other industrial solvents. The screening results indicated that chemical concentrations in most areas were below the ATSDR health guidelines of concern. The EPA stated on its website, “The low level of volatile pollutants is not surprising as contaminants may be bound in sediment. Monitoring data directly around Murphy Oil spill reveal some slightly elevated levels of benzene and toluene that are associated with petroleum release. Long-term exposure (a year or longer) at the levels measured would be required for health effects to be a concern.”

Air may also play a role in an illness known as “shelter cough,” or “Katrina cough.” Shelter cough is presumed to be an allergic reaction to some particulate matter in the air, according to Stephens. However, despite the presence of shelter cough and earlier concerns about a wave of infectious diseases in the wake of Katrina, acute respiratory illness have made up only 8.7% of diagnoses between August 29 and September 24, according to the October 7 *Morbidity and Mortality Weekly Report*. “We have no evidence of infectious disease outbreaks,” Stephens said at the EHSRT.

## A Mountain of Debris

The amount of debris generated by Katrina is by all accounts staggering. FEMA estimates there are 39.9 million cubic yards of debris in Mississippi alone. Mark Williams, administrator of solid waste policy, planning, and grants at the Mississippi Department of Environmental Quality (MDEQ), says that state has enough space for the initial removal of debris to staging areas, but not for long-term deposition in landfills.

Jimmy Guidry, medical director of Louisiana’s Department of Health and Hospitals, says Louisiana, too, lacks sufficient landfill space for all the debris: “We have more than three hundred thousand refrigerators that need to be disposed of. All these have freon in them.” Guidry said at the roundtable that the Louisiana Department of Environmental Quality has approved dozens of temporary debris disposal sites, which will have to be carefully monitored.

Appliances can be recycled for metal content. Televisions and household computers pose a different problem. A single computer monitor contains 4.5 pounds of lead, and computer processing units contain trace metals that can leach out of unlined landfills.

As much as one-third of the debris is vegetative matter that can be burned or chipped for compost. The rest must be recycled or landfilled. Williams says burning of vegetative debris has been allowed in Mississippi for some months and is now largely complete. He adds, “EPA in conjunction with MDEQ has done some monitoring in the area [of controlled burns], which has indicated some elevated levels of formaldehyde and acrolein in certain areas.” In the interest of minimizing air pollution, the EPA and MDEQ allowed only clean vegetative debris to be burned and strongly encouraged the use of air curtain destructors and other combustion units in the early stages of cleanup.

Williams says another daunting challenge was disposing of thousand of tons of food—chicken, fish, and beef—rotting in warehouses on the docks. Officials from Mississippi’s Natural Resources Conservation Service said more than 6 million dead animals—poultry and livestock—had to be removed from farms in the affected area. Now officials are dealing with wastes in homes, including such items as propane tanks, household pesticides, and asbestos from roofing, insulation, and other home sources. The waste is taken to staging areas where hazardous waste is pulled out for disposal by the EPA. As of October 31, the EPA had collected an estimated 1 million pounds of household hazardous waste in Louisiana (the agency did not report on collections in other states).

## Injury Protection

One of the major concerns officials have with regard to the handling and disposal of debris is the safety of workers. “We have a large number of workers coming to the Gulf seeking employment, and many of them are not properly trained and protected,” says Max Kiefer, assistant director of emergency preparedness and response for the National Institute for Occupational Safety and Health (NIOSH). High-risk occupations include debris removal, levee rebuilding, residential refurbishment, and infrastructure rebuilding.

NIOSH is trying to keep workers apprised of health hazards. “We have assessed exposure to silica and metals during levee rebuilding, debris removal, and tasks involving the sediment,” Kiefer said at the roundtable. “We also worried that people were wearing protective gear that may induce heat stress. After assessing certain tasks, we were able to downgrade our gear recommendations in light of that. Psychological stress on responders has been significant. But by far the biggest issue has been injuries—lacerations, falls, and trips.” NIOSH is providing guidance for responders and providers on the CDC hurricane response website.

Private citizens also face significant risk of injury during cleanup. Officials talk of a “second wave” of injury following a natural disaster as citizens undertake to remove debris and repair buildings themselves. Will Service, the industrial hygiene coordinator with the North Carolina Office of Public Health Preparedness and Response, worked in a mobile hospital in Waveland, Mississippi, in the days following the storm. “We saw a lot of injuries from things like chain saws used during cleanup,” Service says. “People are tired, their thinking isn’t clear. They’re doing things they don’t normally do.”

Illnesses and injuries associated with Katrina are being tracked by the CDC, with updates posted regularly on its website. Confirming what public health officials warned about a second wave of injuries, the most common diagnosis (26.2%) in reporting hospitals and clinics from September 8 to October 4 was injury. The major cause of injury was falling, followed closely by vehicle crash–related injuries (likely related to missing or nonfunctioning traffic signs and signals). Cutting and piercing injuries ranked third.

## Coming Home to Hazards

Mold growth in houses damaged by Katrina is of enormous concern to health and housing officials. Estimates of the number of homes suffering water damage range in the hundreds of thousands. Claudette Reichel, an LSU professor of education and housing specialist, says that virtually every home that sustained flood damage will experience mold growth. “Houses that people were not allowed back into for weeks will all have mold, and that mold will have had time to multiply, spread, and get really thick,” she says. Says Frumkin, “The magnitude of mold exposure in the Gulf region will in many instances greatly exceed anything we have seen before, adding to the concern and uncertainty regarding health effects.”

How or even whether mold causes human health problems is disputed by public health professionals, but most acknowledge a connection. “It is a very difficult science, because there is no clear-cut dose–response threshold,” Reichel says. “It is highly dependent upon the type of mold, whether the mold is producing a mycotoxin, the susceptibility of the patient, and the amount of exposure.”

The CDC states that people who are sensitive to mold may experience stuffy nose, irritated eyes, or wheezing. People allergic to mold may have difficulty in breathing. People with weak immune systems may develop lung infections.

Health and housing officials advise homeowners and renters to throw out any furnishings, insulation, and bedding that may have gotten wet, to clean walls and floors with soap and water, to ventilate, and then to close up and dehumidify the home.

The CDC also reported a spike in post-Katrina carbon monoxide poisoning in the Gulf Coast in the October 7 *Morbidity and Mortality Weekly Report*. From August 29 to September 24, a total of 51 cases of carbon monoxide poisoning, including 5 deaths, were reported in Alabama, Louisiana, and Mississippi. After the hurricanes, many residents used gasoline-powered portable generators to provide electricity to their homes and businesses. These devices produce carbon monoxide, which can build up to fatal levels if run inside a living space or garage.

A number of other health issues loom as residents begin returning to New Orleans, where health care services aren’t widely available, sewer and water services are still spotty, and structural inspections aren’t complete. Residents have asked city officials for a health assessment to address their concerns about oil spills, mold contamination, and the possible long-term health effects related to mold and chemical exposures. “We are developing an assessment tool for this purpose, and we anticipate that it will be developed for the beginning of [2006],” says Stephens.

Many health care professionals worry that mental health may be the most serious long-term health issue resulting from Katrina. Hundreds of thousands of people across the Gulf region have had their homes destroyed. Thousands are still living in shelters. Many have no jobs, no health insurance, and no job prospects. “We are seeing a lot of symptoms of post-traumatic stress disorder,” says Marty Allen, a psychologist with the Mississippi Department of Mental Health. “The trauma was not just the day of the storm. People are still being traumatized by living in tents, not having jobs, and having to walk for miles just to get food and water.”

## Lessons Learned?

What lessons have been learned from Katrina with respect to environmental health? Debate about how to protect Gulf Coast citizens from hurricanes and storm surge was ongoing before the storm and will continue with renewed intensity.

In Mississippi, Governor Haley Barbour enlisted the Chicago-based Congress for New Urbanism to come up with recommendations for rebuilding the Gulf Coast. The Congress sponsored a week-long Mississippi Renewal Forum in October attended by some of the nation’s leading architects, engineers, and urban planners. Working with local leaders, the teams produced reports for 11 coastal towns impacted by the storm. Recommendations include improving the connectivity between towns by moving the CSX freight line north and transforming the abandoned right-of-way into a boulevard for cars and transit, connecting the Gulf region towns with high-speed rail, realigning and revising U.S. 90 to become a pedestrian-friendly “beach boulevard,” and creating a Gulf Coast bikeway.

A similar process is under way in Louisiana under the auspices of the Louisiana Recovery Authority created by Governor Kathleen Blanco. The authority is developing short-, medium-, and long-range plans to guide the rebuilding of Louisiana in the wake of the hurricanes. At the authority’s request, the American Association of Architects, in collaboration with the American Planning Association, presented the Louisiana Recovery and Rebuilding Conference on November 10–12. The authority has developed a 100-day plan that includes completion of an environmental evaluation of damages caused by the hurricanes and development of recommendations for how to proceed with reconstruction.

Discussion will center on how to protect New Orleans from further flooding and whether certain low-lying parts of the city should even be reoccupied. Such decisions will be made in the months and years to come. Meanwhile, environmental and public health officials have drawn some conclusions about how to better respond to events like Katrina.

Officials at the EHSRT agreed that communication in advance and in the wake of natural and man-made disasters is key. Fears and rumors of disease ran rampant in the days following Katrina. Citizens, the media, and even public health officials did not know which factors presented a genuine health threat and which did not. Federal agencies conducted testing and provided data, but people often did not know how to interpret those data with respect to the kinds of exposures they were encountering.

“The public health community must be actively involved and articulate key health issues,” said Kellogg Schwab, an assistant professor at Johns Hopkins Bloomberg School of Public Health. “We must keep the message simple and focused. We must develop effective strategies to provide targeted timely results. We must provide concise and accurate public health information and advice.”

Officials also agreed that responders must be properly trained and deployed, provided with proper protective gear and an effective communications system (land lines and cell phones were inoperative in much of the area for weeks after Katrina). Health officials must be able to assess the particular kinds of exposures that people have been subjected to and respond accordingly.

“Your response strategy for exposure varies with each event,” said Paul Lioy, deputy director of the Environmental and Occupational Health Sciences Institute at Rutgers University. “The World Trade Center [collapse] was an instantaneous acute air exposure event like we’d never experienced. Katrina for the most part involved an acute water exposure event, but the exposure was over a longer period of time.”

Lioy pointed out the need for a national review of the kind of standards and guidelines necessary to ensure that the correct information is given out to the public about immediate hazards versus long-term exposures and risks. “Comparison to general drinking water or ambient air quality standards are not sufficient for guiding the public or public officials during an acute exposure event,” he said.

Most of all, roundtable participants agreed, Katrina represents a chance for officials across all levels of government to do things better—evacuation planning, urban design, communication, environmental monitoring, and involvement of citizenry, particularly minority and low-income residents. John McLachlan, director of the Tulane/Xavier Center for Bioenvironmental Research, said that preparing for disasters like Katrina requires the involvement of virtually every academic discipline. To that end, Tulane and Xavier are creating a Katrina Environmental Research and Restoration Network (KERRN) of researchers who share data and ideas across disciplinary, geographical, and institutional lines. Paraphrasing one of his colleagues, McLachlan stated, “This is the mother of all multidisciplinary problems.”

## Figures and Tables

**Figure:**
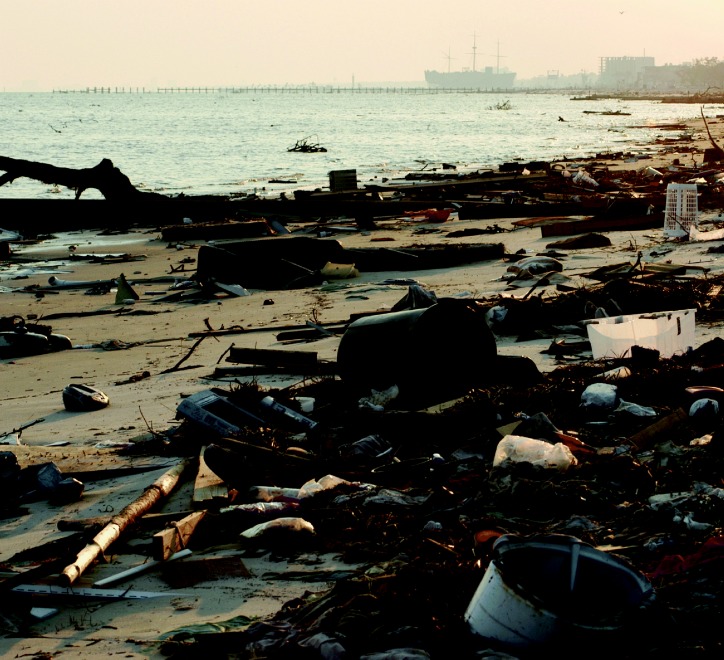


**Figure:**
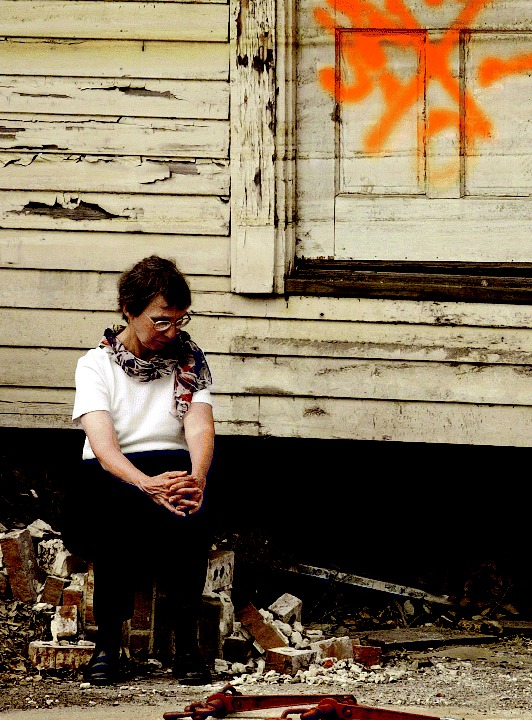
Comprehending the catastrophe. (above) Phyllis Howley, 70, sits on what’s left of the porch of her son’s New Orleans home. (left) The beach in Biloxi, Mississippi, four days after Katrina.

**Figure:**
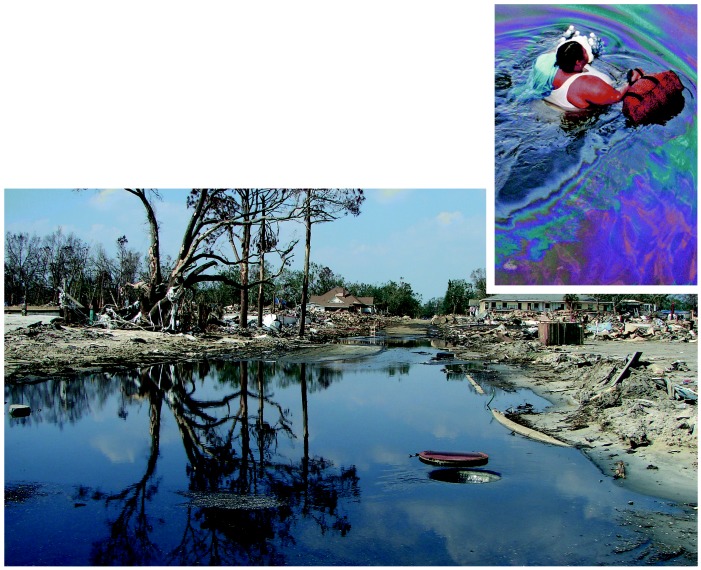
Hazards in wading? Initial reports labeled the floodwaters through which many New Orleans residents were forced to wade a “toxic gumbo.” Later testing of stormwaters found elevated levels of fewer contaminants than feared, but sampling was limited and the water may yet present long-term problems.

**Figure:**
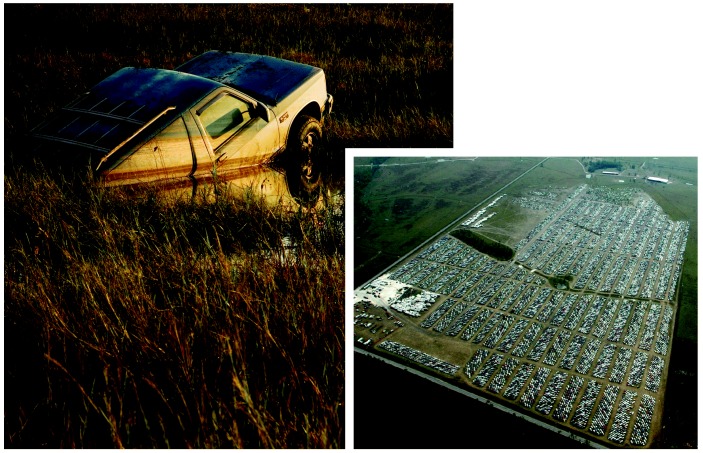
Vehicle slaughter. Vehicles destroyed in the storm surge of Hurricane Katrina (left) are being stockpiled north of Gulfport, Mississippi (right). The thousands of automobiles are just the tip of the iceberg of waste that communities must deal with as a result of the hurricane.

**Figure:**
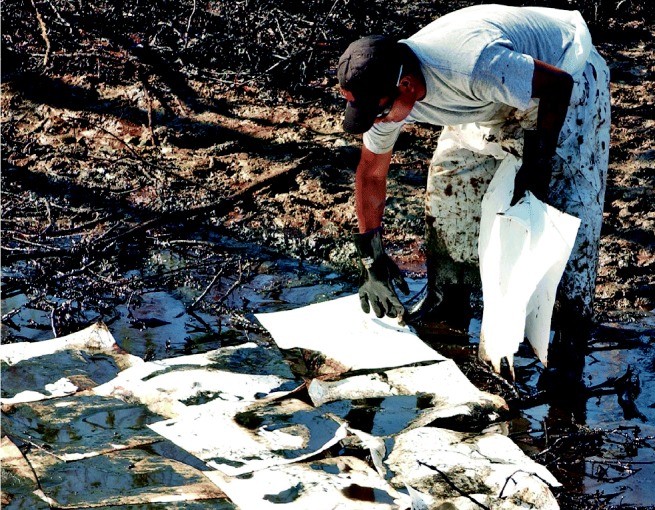
A slicker picker-upper. Absorbent pads are used to clean up surface oil at the Bass Enterprises South Facility in Cox Bay, Louisiana, where Katrina caused the release of an estimated 3.8 million gallons of oil. Oil spills may have long-lasting effects on water supplies and surrounding ecologies.

**Figure:**
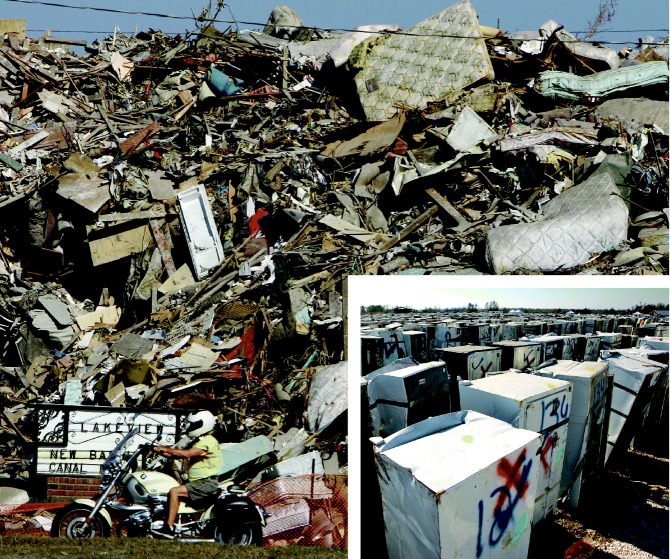
Waves of destruction. (above) A motorcyclist rides past a mountain of trash, wallboard, and furniture removed from homes damaged by Katrina. (inset) Thousands of damaged refrigerators await safe disposal at a landfill near New Orleans. The freon in these appliances will need to be handled carefully.

**Figure:**
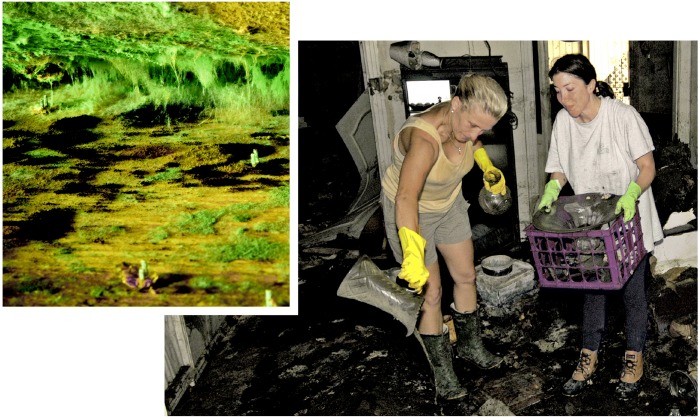
Opportunistic attacker. The warm, damp conditions left in homes following Katrina provided the perfect medium for the growth of mold. Because mold can be extremely toxic and hard to eradicate, many homes may not be salvageable.

**Figure:**
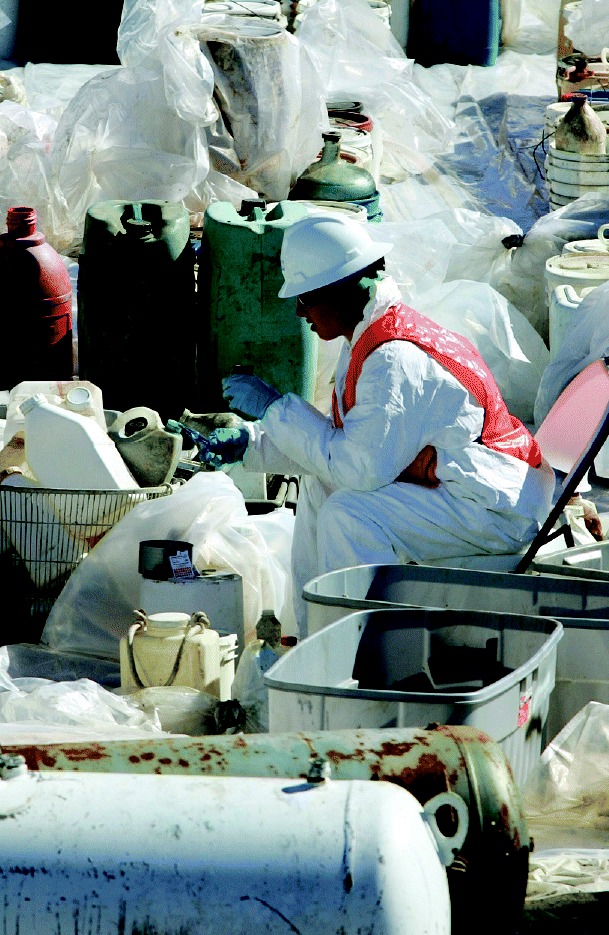
Chemical calamity. A worker tests hazardous household liquids at the Fort Jackson “orphan” tank and drum staging area in Louisiana.

